# Brown syndrome with severe amblyopia: a case report from Africa

**DOI:** 10.11604/pamj.2015.20.56.6050

**Published:** 2015-01-21

**Authors:** Bolutife Ayokunnu Olusanya

**Affiliations:** 1 Department of Ophthalmology, University College Hospital, Ibadan, Nigeria

**Keywords:** Brown syndrome, amblyopia, Africa, child, squint, eye movement

## Abstract

Brown syndrome is a rare form of strabismus that is characterised by restriction of elevation of the eye in adduction. This is a case report of an eleven year old Nigerian girl who presented with a history of squint and poor vision. She had visual acuities of 6/24 and counting fingers in her right and left eyes respectively. There was a left hypotropia in the primary position of gaze with associated marked restriction of elevation in adduction and a positive forced duction test. Refraction revealed a refractive error of +4.50 diopters in each eye. The right visual acuity improved significantly to 6/9 with the refractive correction while the left visual acuity improved marginally to 6/60. This report demonstrates the occurrence of Brown syndrome with associated severe amblyopia in Africa. Health care providers are encouraged to promptly refer all patients, especially children, who have ocular motility disorders for early specialist intervention.

## Introduction

Brown syndrome is an uncommon ocular motility disorder characterised by restriction of elevation of the adducted eye [1]. It is the most frequent cause of an apparent isolated palsy of the inferior oblique muscle [2], one of the extra-ocular muscles responsible for ocular motility. Brown syndrome occurs when there is a mechanical restriction of the superior oblique muscle tendon which leads to difficulty in moving the affected eye upwards, particularly in the adducted position. Essentially, the oblique muscles have opposing actions in moving the eyes in the vertical plane. The action of the inferior oblique muscle is to move the eyeball upward and inward (with the eye looking towards the glabella), while the superior oblique muscle moves the eyeball downward and inward (to look towards the nostril on the same side). Normally, when the inferior oblique contracts, the superior oblique must relax to allow the eye to move into the field of action of the inferior oblique. If the superior oblique tendon is shorter than normal or cannot slide freely, as occurs in Brown syndrome, the affected eye would not be able to elevate in adduction. This rare form of strabismus was first described by Harold W. Brown in 1950 [3]. The syndrome may be congenital or acquired. Majority of cases are congenital, while the acquired type is usually secondary to either trauma or inflammation [4]. Most of the time, Brown syndrome occurs in a sporadic fashion and is unilateral, but it may be bilateral in 10% of cases [1]. There is no definite gender predisposition and there is no predilection for the right or the left eye [1].

Amblyopia is an ocular disorder characterised by poor vision that persists in spite of the use of appropriate spectacle correction coupled with the absence of any structural abnormality of the visual pathways that can explain such visual impairment [5]. It generally occurs as a result of inadequate or abnormal visual inputs during the sensitive period of visual development (i.e. from birth to the age of seven years. To the best of the author's knowledge, there are no prior reports about the occurrence of Brown syndrome in Africa. Furthermore, severe amblyopia which has not been commonly associated with the syndrome. This case reports highlights the fact the amblyopia can occur as a sequela of congenital Brown syndrome especially when it is diagnosed in late childhood or adulthood.

## Patient and observation

**Figure 1:**
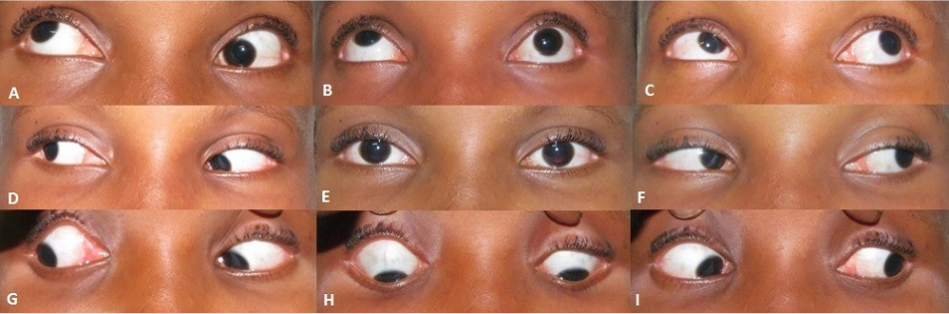
A composite picture showing the nine position of gaze. 1A shows the marked limitation of elevation of the left eye when she tried to look up to the right, while 1C shows full elevation of the left eye when looking up to the left

A four year old girl presented to the paediatric eye clinic of the University College Hospital, Ibadan, Nigeria with a history of squint and poor vision. Her mother had noticed a misalignment of the eyes three years previously and the patient had been complaining about poor distance vision for about a year. There was no history of antecedent trauma to the head or face. There was no history of double vision (diplopia) or pains on ocular movements. There was no associated headache, fever, vomiting, joint pains or skin rashes/ lesions. The review of systems was essentially normal and she had enjoyed good health since early childhood. Her pregnancy and delivery were uneventful; and her development was normal. All other family members were well and there was no family history of squint. Unaided visual acuity was 6/24 in the right eye and counting fingers (CF) in the left eye. During refraction, her visual acuity improved to 6/9 and 6/60 in the right and left eyes respectively. The refractive error detected was a hypermetropia of +4.50 diopters in each eye. Examination revealed normal anterior and posterior segments bilaterally. There was about 10 prism diopters of left hypotropia with no horizontal deviation in the primary position (Figure 1) and she did not have an abnormal head posture. Her palpebral fissures were normal and equal on both sides in the primary position of gaze. Examination of the ocular motility elicited marked limitation of elevation of the left eye when looking to the right, while elevation of the same eye was almost full on left gaze (Figure 1). The movements of right eye were normal and there were no significant changes in the palpebral fissures of both eyes during eye movements. Forced duction test (performed under sedation) was positive and revealed marked restriction of passive elevation of the left eye in adduction. Her systemic examination was completely normal.

A diagnosis of congenital left Brown syndrome with severe amblyopia was made. Spectacles were prescribed and she was commenced on a trial of amblyopia therapy which consisted of patching of the right eye for a period of 3-4 hours every day (after school). Her mother was counselled and educated about the condition as well as the treatment plan. Specifically, she was informed that surgery was necessary to correct the squint, irrespective of the outcome of the amblyopia therapy. A three month follow up visit was scheduled, however, the patient defaulted after the initial visit.

## Discussion

When Brown initially reported this ocular motility disorder, he noted the following clinical features: slight downward deviation of the affected eye on adduction; limitation of elevation on adduction; widening of the palpebral fissure on adduction; absence of over-action of the ipsilateral superior oblique; V pattern exotropia; and a positive forced duction test [6]. This patient exhibits these features except horizontal deviation and palpebral fissure changes. Notwithstanding, the presence of two features, specifically the limitation of elevation in adduction and the positive forced duction test are strongly suggestive of a diagnosis of Brown syndrome. These two features have been described as consistent features of Brown syndrome [7]. The main differential diagnosis that must be considered in this patient is an isolated left inferior oblique paralysis [8]. This very rare condition has some similar features to BS, in particular, the limitation of elevation in adduction. The positive forced duction test, however, is the major distinguishing feature. Inferior oblique paralysis is principally characterised by a negative forced duction test, in which there is no resistance to the passive movement of the eyeball into the direction of action of the inferior oblique muscle. In addition, there is usually associated over-action of the superior oblique muscle, which was absent in this patient. Another differential diagnosis is congenital fibrosis of the inferior rectus muscle. This condition, however, usually causes restriction of elevation of the eye in abduction as well as in adduction, unlike in Brown syndrome [7].

The absence of a history of squint since birth appears to be indicative of an acquired form of Brown syndrome in this patient. The congenital type is, however, more likely, for the following reasons. Firstly, the acquired Brown syndrome is relatively uncommon in childhood and is mostly reported in adult patients [6]. Secondly, the severity of the amblyopia is suggestive of a longstanding disturbance of the visual inputs of the left eye during the period of visual development. Thirdly, most cases of acquired Brown's syndrome are due to trauma or inflammation of the superior oblique tendon/trochlea complex. Acquired Brown syndrome has been associated with juvenile chronic arthritis [9], rheumatoid arthritis [10, 11], Systemic lupus erythematousus [2, 12], scleroderma [13], primary Sjogren's syndrome [8], Hurler-Scheie's syndrome [14], repair of medial orbital wall fractures [15, 16] and orbital metastasis [17]. It may also be caused by a surgery to correct paralysis of the superior oblique tendon [14]. There were no symptoms or signs to suggest any of these causes in this patient. The occurrence of severe amblyopia in this patient with Brown syndrome is remarkable. Previous reports have indicated that amblyopia occurs infrequently in Brown syndrome [1, 7, 18]. A few authors, however, have laid some emphasis on the possibility of amblyopia in Brown syndrome patients [19, 20]. Suppression is said to be rare in Brown syndrome [7]. Yet, in this case, the nonexistence of diplopia and an abnormal head posture in the presence of the left hypotropia support the likelihood that the visual inputs from the left eye had undergone long-term suppression which led to amblyopia in that eye.

## Conclusion

This case report demonstrates that severe amblyopia may be associated with Brown syndrome in Africa. There is a significant probability that the amblyopia found in this patient would have been less severe if the condition had been diagnosed earlier. It is therefore vital to educate health care providers to promptly refer patients, especially children, with ocular motility disorders to ensure early diagnosis and treatment.
